# Comparative and functional genomics of the *Lactococcus lactis* taxon; insights into evolution and niche adaptation

**DOI:** 10.1186/s12864-017-3650-5

**Published:** 2017-03-29

**Authors:** Philip Kelleher, Francesca Bottacini, Jennifer Mahony, Kieran N. Kilcawley, Douwe van Sinderen

**Affiliations:** 1 School of Microbiology, University College Cork, Cork, Ireland; 2 Alimentary Pharmabiotic Centre, University College Cork, Cork, Ireland; 3 Department of Food Biosciences, Teagasc Food Research Centre, Moorepark, Cork, Ireland

**Keywords:** *Lactococcus lactis*, Genomics, SMRT sequencing, Pan-genome, Niche adaptation

## Abstract

**Background:**

*Lactococcus lactis* is among the most widely studied lactic acid bacterial species due to its long history of safe use and economic importance to the dairy industry, where it is exploited as a starter culture in cheese production.

**Results:**

In the current study, we report on the complete sequencing of 16 *L. lactis* subsp. *lactis* and *L. lactis* subsp. *cremoris* genomes. The chromosomal features of these 16 *L. lactis* strains in conjunction with 14 completely sequenced, publicly available lactococcal chromosomes were assessed with particular emphasis on discerning the *L. lactis* subspecies division, evolution and niche adaptation. The deduced pan-genome of *L. lactis* was found to be closed, indicating that the representative data sets employed for this analysis are sufficient to fully describe the genetic diversity of the taxon.

**Conclusions:**

Niche adaptation appears to play a significant role in governing the genetic content of each *L. lactis* subspecies, while (differential) genome decay and redundancy in the dairy niche is also highlighted.

**Electronic supplementary material:**

The online version of this article (doi:10.1186/s12864-017-3650-5) contains supplementary material, which is available to authorized users.

## Background

*Lactococcus lactis* is a Gram positive, catalase-negative, non-motile and coccoid bacterium [1]. *L. lactis* has a long history of safe use in the fermented food industry and as such enjoys a so-called “GRAS” (Generally Regarded as Safe) status. Lactococcal strains are particularly important to the dairy industry, where they are employed as starter cultures for cheese production. *L. lactis* has four component subspecies, two of which are routinely employed in the dairy fermentation sector, i.e. subspecies (subsp.) *cremoris* and subsp. *lactis* (and a biovariant; subsp. *lactis* biovar diacetylactis, which distinguishes itself based on citrate metabolism, see also below). The two remaining *L. lactis* subspecies, i.e. *L. lactis* subsp. *hordniae* isolated from the leafhopper *Hordnia circellata* [2], and *L. lactis* subsp. *tructae* isolated from brown trout, *Salmo trutta* [3], are considerably under-represented in both biological and genomic studies compared to their dairy-associated counterparts.

Genetically, a typical *L. lactis* chromosome ranges in size from ~2.2 to 2.6 Mb, often accompanied by a rich plasmid complement [4] and multiple integrated (remnant) prophages [5]. Reductive evolution and genome decay have previously been reported in ‘domesticated’, dairy *L. lactis* strains, particularly those belonging to subspecies *cremoris* [6, 7]. Niche adaptation by lactococcal strains has been investigated most thoroughly in relation to the dairy environment. In this particular niche, strain adaptations appear to be mainly plasmid-encoded and two examples of this are lactose and citrate utilisation. Lactose utilisation in *L. lactis* is performed via the *lac* operon, which consists of the *lacABCDEFGX* genes and which is regulated by the repressor *lacR* [8, 9]. Citrate metabolism by citrate-positive (Cit^+^) lactococci is mediated by the *citQRP* operon [10]. The classification of Cit^+^ lactococci as *L. lactis* subsp. *lactis* biovar diacetylactis has led to confusion as plasmid-encoded characteristics can be transferred from one strain to another and may lead to incorrect classification based on phenotype [11], highlighting the importance of genome sequencing for the correct characterisation of members of this taxon.

The advent of modern sequencing technologies has made whole genome analysis more accessible, and as a result there are now 84 lactococcal assemblies publicly available in the NCBI (National Centre for Biotechnology Information) database, 14 of which represent complete genome sequences including the two prototypical stains *L. lactis* subsp. *lactis* IL1403 [12] and *L. lactis* subsp. *cremoris* MG1363 [13]. To date a number of comparative genome studies have been conducted and have provided novel insights into lipolysis [14], prophage [5, 6], proteolysis [15], taxonomy [16] and niche adaptation functions of these strains [17].

In the current study we applied one of the latest sequencing technologies, Single-Molecule-Real-Time (SMRT) sequencing developed by Pacific Biosciences [18, 19] to contribute a further 16 complete lactococcal genomes to the public database. The increased dataset of complete lactococcal genomic sequences allows for the investigation of the corresponding pan-genome, which when closed defines the total number of genes encoded in the *L. lactis* taxon [20–22]. Furthermore, phylogeny, core and non-core genes, metabolism and niche-specific adaptations in terms of the total genetic content of the taxon were examined.

## Results

### General genome features

In this study, the chromosomal features of 30 *L. lactis* strains were assessed, 18 of which belong to subspecies *lactis* and a further 12 to subspecies *cremoris* based on phylogenetic analysis of 16S RNA. For all selected strains, complete genome assemblies were available, of which 14 were obtained from the NCBI (National Centre for Biotechnology Information) database, while the remaining 16 were sequenced as part of the current study using the SMRT sequencing approach (Table 1). Although the NCBI database contains in total 84 *L. lactis* genome assemblies, only those, which are fully finished (i.e. present in the data base as a single chromosomal contig), were selected for this project due to the inherent limitations of draft assemblies. Briefly, the order and orientation of contigs of such draft assemblies remains unresolved and the differentiation between traits, which are verified to be chromosomally-encoded versus plasmid-encoded, is not possible particularly when one considers plasmid integration events. Most notable, however, is the finite nature of a finished genome which facilitates the comparison of the full genetic content of a strain rather than most of the genetic content, whereas in the case of a draft genome the likelihood of error from missing genes or incorrect copy number is significantly higher [23, 24].

**Table 1:** Lactococcal representative strains used in this study

Strain name	Genbank accession	Ecological niche	Sequencing technology	Year	Citation
subsp. *lactis*					
Il1403	AE005176	Dairy isolate	Sanger	2001	[12]
KF147	CP001834	Plant isolate	454-pyrosequencing & Illumina	2009	[65]
CV56	CP002365	Human isolate	454-pyrosequencing	2011	[66]
IO-1	AP012281	Drain water	Sanger	2012	[67]
KLDS 4.0325	CP006766	Koumiss	Illumina	2013	[68]
NCDO 2118	CP009054	Frozen peas	SOLiD, Ion PGM & Ion Torrent PGM	2014	[69]
SO	CP010050	Dairy isolate	Ion Torrent PGM	2014	[70]
AI06	CP009472	Açaí palm	454-pyrosequencing	2014	[26]
184	CP015895	Dairy isolate	PacBio SMRT	2016	**
229	CP015896	Dairy isolate	PacBio SMRT	2016	**
275	CP015897	Dairy isolate	PacBio SMRT	2016	**
UC06	CP015902	Dairy isolate	PacBio SMRT	2016	**
UC08	CP015903	Fermented meat	PacBio SMRT	2016	**
UC11	CP015904	Fermented meat	PacBio SMRT	2016	**
UC063	CP015905	Dairy isolate	PacBio SMRT	2016	**
UC77	CP015906	Dairy isolate	PacBio SMRT	2016	**
UL8	CP015908	Dairy isolate	PacBio SMRT	2016	**
C10	CP015898	Dairy isolate	PacBio SMRT	2016	**
subsp. *cremoris*					
SK11	CP000425	Dairy isolate	Sanger	2006	[7]
MG1363	AM406671	Dairy isolate	Sanger	2007	[13]
NZ9000	CP002094	Laboratory strain	Illumina	2010	[71]
A76	CP003132	Dairy isolate	Sanger	2011	[72]
UC509.9	CP003157	Dairy isolate	454-pyrosequencing & Illumina	2012	[6]
KW2	CP004884	Dairy isolate	454-pyrosequencing	2013	[73]
158	CP015894	Dairy isolate	PacBio SMRT	2016	**
UC109	CP015907	Dairy isolate	PacBio SMRT	2016	**
JM1	CP015899	Dairy isolate	PacBio SMRT	2016	**
JM2	CP015900	Dairy isolate	PacBio SMRT	2016	**
JM3	CP015901	Dairy isolate	PacBio SMRT	2016	**
JM4	CP015909	Dairy isolate	PacBio SMRT	2016	**

** Sequenced in the framework of this study

The 30 *L. lactis* strains included in this study encompass six different ecological niches; dairy, plant, meat, fermented foods, human isolate (this is a vaginal isolate of a healthy woman) and a strain isolated from a sink drain, with the vast majority isolated from the dairy environment, most notably for the production of cheese (Table 1). Comparison of the 30 lactococcal genomes established an average chromosome length of 2.428 Mbp from a range of 2.250–2.589 Mbp, where it should be noted that in general the genomes of subsp. *lactis* are larger than their subsp. *cremoris* counterparts (Table 2). Genomes belonging to the subsp. *cremoris* contain a higher proportion of pseudogenes and insertion sequence (IS) elements/transposons, indicative of transpositions and (associated) genome decay within the subsp. *cremoris* genome. A defining characteristic of both subspecies is evident in the number of plasmids within each strain. *L. lactis* carries many niche-specific adaptations within its plasmid complement, particularly for the dairy environment, such as lactose utilisation and casein utilisation, and this is evident in the larger plasmid complement observed for subsp. *cremoris* strains predominantly isolated from the dairy niche. A substantial proportion of the observed genomic diversity is due to a variable number of integrated prophage elements (Table 2).

**Table 2:** General genome features of thirty representative *L. lactis* genomes

Strain	Genome length (Mbp)	CDS	tRNA features	rRNA features	Hypothetical proteins %	Assigned function %	Pseudo genes	IS elements/transposases	Prophage	Plasmids	GC %
*L. lactis* subsp. *lactis*											
184	2343	2312	51	15	19.6	80.4	15	59	2 In ^a^ 6 Re ^b^	3	35.16
229	2455	2541	56	15	20.2	79.8	15	94	4 In 3 Re	5	35.19
275	2496	2418	58	18	20.2	79.8	14	43	3 In 6 Re	4	35.49
UC06	2571	2472	61	18	21.7	78.3	8	35	2 In 3 Re	3	35.26
UC08	2382	2246	62	18	20.0	80.0	14	18	2 Re	3	35.00
UC11	2382	2237	60	19	20.0	80.0	16	17	2 Re	6	35.00
UC063	2393	2361	59	18	19.2	80.8	14	59	3 In 5 Re	5	35.32
UC77	2538	2541	66	21	19.0	81.0	12	96	5 In 3 Re	2	35.26
UL8	2422	2405	59	17	18.5	81.5	13	56	3 In 7 Re	3	35.29
C10	2336	2294	50	15	17.7	82.3	21	53	5 In 3 Re	1	35.30
IL1403	2366	2267	62	18	21.0	79.0	43	43	3 In 3 Re	-	35.33
KLDS 4.0325	2589	2587	64	19	34.0	66.0	56	39	4 In 7 Re	-	35.36
NCDO 2118	2555	2334	66	19	28.0	72.0	52	16	2 In 3 Re	1	34.91
KF147	2598	2537	68	19	19.5	80.5	93	29	2 In 4 Re	1	34.91
SO	2489	2281	64	19	21.5	78.5	126	45	3 In 3 Re	-	35.23
AI06	2398	2197	61	19	22.9	77.1	2	5	1 In 1 Re	-	35.04
CV56	2399	2301	62	19	23.7	76.3	51	31	2 In 4 Re	5	35.24
IO-1	2422	2233	65	18	23.1	76.9	8	13	1 In 1 Re	-	35.10
Average: (*lactis*)	2451	2364	60	18	21.6	78.4	31	41	3 In 4 Re	2.3	35.18
*L. lactis* subsp. *cremoris*											
158	2250	2078	60	19	17.9	81.1	106	150	2 Re	6	35.88
UC109	2248	2081	60	19	20.0	80.0	98	149	2 Re	6	35.91
JM1	2397	2308	60	19	20.5	79.5	74	243	1 In 6 Re	7	36.01
JM2	2374	2316	58	19	19.6	80.4	68	167	1 In 3 Re	4	35.80
JM3	2454	2411	59	19	23.7	76.3	60	163	2 In 3 Re	5	35.87
JM4	2380	2293	60	19	20.9	79.1	88	181	1 In 4 Re	5	35.83
UC509.9	2250	1947	60	19	18.5	81.5	182	125	1 Re	8	35.88
SK11	2439	2390	61	20	26.2	73.8	144	159	2 In 3 Re	5	35.86
A76	2453	2643	57	19	25.8	74.2	193	198	2 In 7 Re	4	35.88
KW2	2427	2268	61	19	20.8	79.2	- ^c^	3	1 In	-	35.74
MG1363	2530	2516	62	7	30.8	69.2	81	60	2 In 4 Re	1	35.75
NZ9000	2530	2514	65	19	35.3	64.7	99	66	2 In 5 Re	-	35.74
Average: (*cremoris*)	2394	2323	60	18	23.3	76.6	100	138	1 In 3 Re	4.25	35.84
Average: (*lactis & cremoris*)	2428	2344	60	18	22.3	77.6	59	80	2 In 4 Re	3.1	35.45

^a^ In: Complete intact prophage

^b^Re: Partial/remnant prophage

^c^ Pseudogenes are not specifically indicated

General feature extractions conducted on each of the chromosomes generated an overall average of 2344 predicted CDS (Coding Sequences) per chromosome form a range of 1947 to 2643 CDSs of which 77.6% can be functionally assigned using BLAST (Basic Local Alignment Search Tool) based on *in silico* predictions, while the remaining 22.4% are assigned as hypothetical proteins (Table 2).

### Phylogenetic analysis and genome synteny

**Fig. 1:**
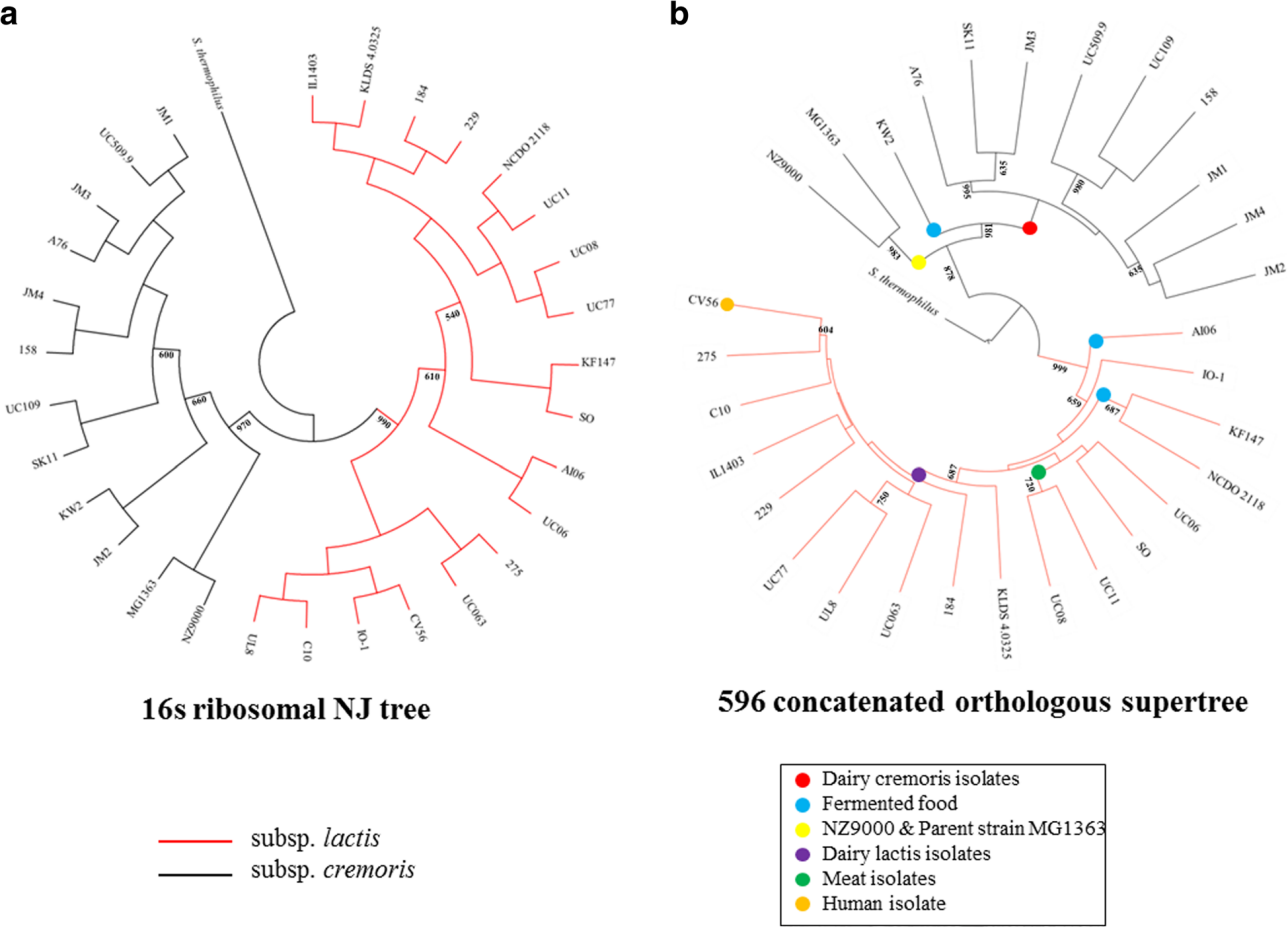
Phylogenetic analysis of *L. lactis* taxon. **a** 16S neighbour-joining (NJ) tree, resulting from the alignment of the 16S rRNA-encoding genes of 30 *L. lactis* isolates, bootstrapped x 1000 replicates, values > 500 are indicated. The corresponding 16S rRNA-specifying sequence of *Streptococcus thermophilus* LMG 18311 was used as an outgroup. **b** Multilocus supertree resulting from the alignment of 596 orthologous genes selected from the core genome, bootstrapped x 1000 replicates, values > 500 are indicated. Ecological niche of representative clades is also indicated

To investigate the phylogenetic relationship between the selected lactococcal isolates, a multifaceted approach was employed. Firstly, the 30 genomes were aligned based on 16S rRNA sequences with *Streptococcus thermophilus* used as an out-group to root the phylogenetic tree, resulting in a clear division into two major clades that correspond to the subsp. *lactis* and subsp. *cremoris* division (Fig. 1a). In order to improve the phylogenetic resolution of the analysis, a second approach was employed by constructing a phylogenetic supertree of 596 conserved orthologous proteins using an approach that has previously been applied to other species [22, 25]. The conserved orthologues were selected based on all-against-all reciprocal BLASTP analysis with an e-value cut-off of 0.0001 and MCL (Markov Clustering) in order to identify single-copy genes conserved across all 31 (30 *L. lactis* plus *S. thermophilus* out-group) genomes in the phylogenetic analysis (Fig. 1b). The generated supertree displays the same bifurcation observed for the 16S rRNA analysis (Fig. 1a & b), substantiating this clear genomic differentiation between the two subspecies. The most notable difference in the lactococcal supertree was that the majority of subclades correspond to niche specificity. Dairy isolates of subsp. *cremoris* cluster together into one clade, distinct from *L. lactis* KW2 isolated from fermented corn, while *L. lactis* NZ9000 and its parent strain *L. lactis* MG1363, which originated from the dairy niche formed their own clade. Dairy isolates of subsp. *lactis* also grouped together, with the exception of *L. lactis* UC06 and *L. lactis* SO. Furthermore, subsp. *lactis* isolates from meat and fermented foods, each formed separate clades (Fig. 1b).

The deduced tree is also indicative of a unique allelic type for CDSs from subsp. *lactis* isolates in comparison to those from subsp. *cremoris* isolates, and is in agreement with the described differences in average nucleotide identity and tetranucleotide frequency correlation coefficients between the two subspecies [16]. To investigate the unique allelic types of the two subspecies, a subset of individual highly conserved housekeeping genes, i.e. those required for the maintenance of basic cellular functions, from each of the genomes were aligned independently (involving the following genes: *recX*, *ssbA*, *recQ*, *radA*, and *radC* [Additional file 1]), resulting in a clear subspecies division in each instance.

To assess the synteny of the lactococcal genomes, whole genome nucleotide alignments were performed and were represented as a dotplot matrix (Additional file 2). *L. lactis* subsp. *lactis* 184 was used as a representative strain for the subspecies, first aligned against itself and then against the remaining 17 subsp. *lactis* genomes. This approach was also employed for the subsp. *cremoris* genomes using *L. lactis* subsp. *cremoris* 158 as the representative strain. Genome synteny was conserved in the *lactis* subspecies with the exception of the *L. lactis* subsp. *lactis* AI06 chromosome, which revealed a large inversion between coordinates 900 Kbp and 1633 Kbp as previously reported [26].

Genome synteny was significantly less conserved among the subsp. *cremoris* strains, with in particular *L. lactis* subsp. *cremoris* strains A76, JM1, JM2, MG1363 and NZ9000 presenting with multiple chromosomal inversions. In the case of genomes sequenced within the scope of this study (by SMRT sequencing, which generates long individual reads; average ~8 Kbp), these observed inversions are assumed to be genuine inversions rather than assembly errors. Visual inspection of the SMRT assembly at points intersecting these inversions allowed for the identification of reads spanning across the point of inversion in each case. The increased incidence of chromosomal inversions within these genomes correlates with the observed high number of transposons and other mobile elements (being 198, 243, 167, 71 and 66, respectively) (Table 2). The suspected role of mobile genetic elements in promoting chromosomal inversions was corroborated by sequence inspection of the borders of each of the identified inverted regions, which revealed in all incidences the presence of multiple transposable elements or integrated prophage(s) (data not shown).

### Pan/core-genome analysis

**Fig. 2:**
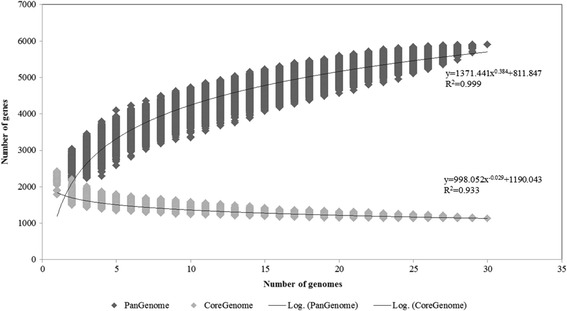
Pan-genome and core-genome of *L. lactis.* Represents accumulated number of new genes in the *L. lactis* pan-genome plotted against the number of genomes added and the accumulated number of genes attributed to the core-genome plotted against the number of added genomes. The deduced mathematical functions are also indicated

To evaluate current sequencing efforts of the *L. lactis* taxon and to determine if additional genome sequencing is necessary to provide a complete overview of the chromosomal diversity of this taxon, pan-genome analysis was applied using the PGAP v1.0 pipeline [27]. The analysis was applied to the chromosomes of *L. lactis* only and excluded plasmid sequences as the main aim of this paper is to report on chromosomal diversity rather than mobile genetic elements. The resulting graph (Fig. 2) reveals an asymptotic curve increasing at an average rate of 209.44 genes for the first 11 chromosomes analysed. Beyond this point, the rate of pan-genome increase slows to an average of 86 genes per genome added for the remaining 19 strains in the analysis resulting in a pan-genome constituted by 5906 genes. The majority of new genes added at this point in the analysis are short hypothetical coding sequences (CDSs) which do not contribute greatly to our current understanding of the genetic diversity of these strains. The deduced mathematical function is also displayed (Fig. 2) and the exponential value (<0.5) indicates that the pan-genome is in a closed state [20].

Using the approach outlined above, it was also possible to deduce the core genome size (Fig. 2) of 1129 genes. Conversely, when the subspecies are separated and the analysis repeated, the core genome size increases to 1406 genes for subsp. *cremoris* and 1413 genes for subsp. *lactis* (data not shown), thus indicating that 277 and 284 additional genes are conserved in all subp. *cremoris* and subp. *lactis* strains, respectively, although not exclusively confined to either species. In the course of this analysis the previously published genomes which were incorporated were not re-annotated as comparison of the genomes sequenced in this study with those previously published indicated that the gene calling and identified ORFs within each of the genomes were largely identical and did not represent significant enough differences to bias the analysis. On completion of the core genome, the variable regions were examined by DNA based alignments utilising BLASTN to ensure that ORFs were absent as a result of missing DNA as opposed to differences in gene calling.

Overall, both analyses show that *L. lactis* contains an essentially closed pan-genome (excluding the plasmid complement) and that there are a sufficient number of strains included in this study to describe the complete genetic repertoire of the taxon.

### Comparative analysis of orthologous genes

**Fig. 3:**
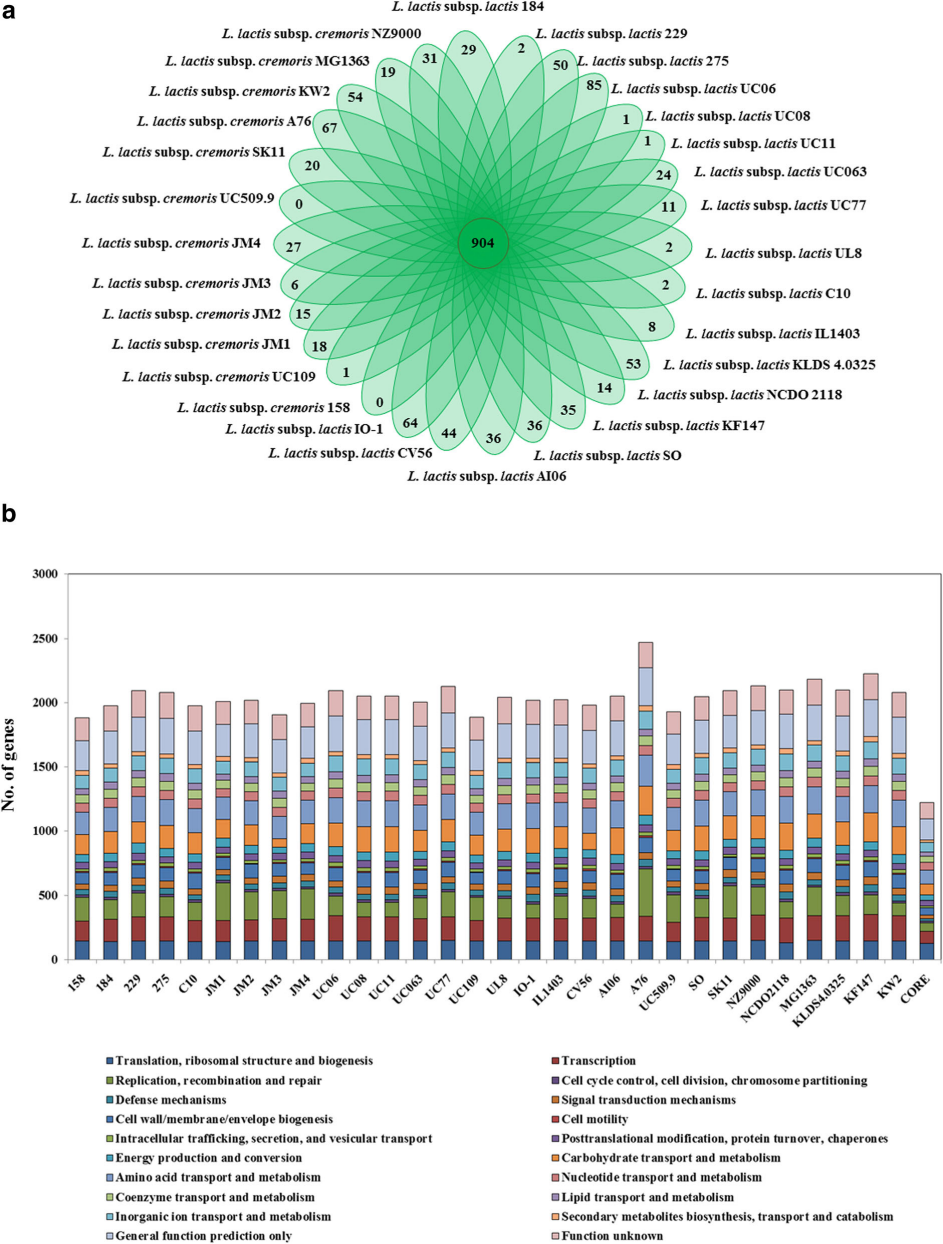
Comparative genomics of orthologous protein groups. **a** Venn diagram displaying core gene families obtained by MCL clustering, and unique genes of 30 *L. lactis* isolates. **b** Cluster of Orthologous Groups (COGs) classification of *L. lactis*. Histograms represent COG predictions for the complete genomes of: *L. lactis* 158, *L. lactis* 184, *L. lactis* 229, *L. lactis* 275, *L. lactis* C10, *L. lactis* JM1, *L. lactis* JM2, *L. lactis* JM3*, L. lactis* JM4, *L. lactis* KF147, *L. lactis* KLDS 4.0325, *L. lactis* KW2, *L. lactis* MG1363, *L. lactis* NCDO 2118, *L. lactis* NZ9000, *L. lactis* SK11, *L. lactis* SO, *L. lactis* UC06, *L. lactis* UC08, *L. lactis* UC11, *L. lactis* UC063, *L. lactis* UC77, *L. lactis* UC109, *L. lactis* UC509.9, *L. lactis* A76, *L. lactis* AI06, *L. lactis* CV56, *L. lactis* IL1403, *L. lactis* IO-1 and the *L. lactis* core genome

To assess the level of (functional) diversity within the lactococcal core and dispensable genomes, comparative analysis was performed via all-against-all, bi-directional BLASTP alignment, and clustering implemented in the MCL pipeline [28, 29]. The core genome of 1129 genes, as defined above, was found to comprise 904 orthologous (single copy) gene families and 225 paralogous (multi-copy) gene families. Gene families unique to each chromosome were also calculated (Fig. 3a) and totalled 757 unique gene families across the 30 assessed *L. lactis* isolates. BLASTP analysis showed that 65% of these unique or dispensable gene families encode proteins of unknown function, while a further 16% encode phage proteins acquired through the integration of a particular prophage-like element. The remaining unique gene families were predominantly found to be representing plasmid integration events encoding proteins involved in mobilisation and conjugation, integrated mobile elements such as transposases and IS elements, or systems that provide specific benefit to the bacterium such as restriction-modification systems, bacteriocin production, and sugar transport and metabolism (Additional file 3).

Cluster of Orthologous Group (COG) analysis was employed to further classify both the core and dispensable genome of *L. lactis*. The 30 lactococcal chromosomes analyzed in this study were classified using COG analysis. The core genome was predominantly composed of genes involved in housekeeping functions, fundamental to growth and survival, while 24% of the genes contained in the core genome were assigned to COG groups [R] and [S] representing genes, for which a general function was predicted or which are of unknown function (Fig. 3b).

COG classification was also performed on the non-overlapping parts of the core genomes of subsp. *cremoris* and subsp. *lactis*, thus focusing on conserved features that differentiate the two subspecies (Table 3). This analysis identified CDSs predicted to be involved in metabolism, particularly transport and metabolism of carbohydrates (Table 3) as the major discerning factor between the two subspecies. Further examination of these subspecies-specific, conserved gene set demonstrates that subsp. *lactis* conserved more unique genes than subsp. *cremoris*, particularly related to metabolism, 124 compared to 68, respectively. The reduced number of CDSs encoding products related to metabolism in subsp. *cremoris* strains is noteworthy as it is in agreement with the generally observed reduced metabolic capabilities of subsp. *cremoris* strains, and highlights the reductive pressure and genome decay imposed on these strains predominantly isolated from the dairy niche.

**Table 3:** COG classifications of the core genomes of *L. lactis*, *L. lactis* subsp. *lactis* and *L. lactis* subsp. *cremoris*

COG classification		Unique core genomes	
	*L. lactis* core genome	*L. lactis* subsp. *lactis*	*L. lactis* subsp. *cremoris*
Translation, ribosomal structure and biogenesis	10%	**<1%**	**5%**
Transcription	8%	**11%**	**9%**
Replication, recombination and repair	6%	**3%**	**6%**
Cell cycle control, cell division, chromosome partitioning	1%	<1%	<1%
Defence mechanisms	1%	4%	3%
Signal transduction mechanisms	2%	2%	2%
Cell wall/membrane/envelope biogenesis	5%	4%	4%
Cell motility	<1%	1%	1%
Intracellular trafficking, secretion, and vesicular transport	1%	<1%	2%
Posttranslational modification, protein turnover, chaperones	4%	1%	<1%
Energy production and conversion	4%	**4%**	**3%**
Carbohydrate transport and metabolism	7%	**14%**	**10%**
Amino acid transport and metabolism	9%	**15%**	**5%**
Nucleotide transport and metabolism	5%	1%	1%
Coenzyme transport and metabolism	4%	2%	2%
Lipid transport and metabolism	3%	3%	3%
Inorganic ion transport and metabolism	6%	4%	3%
Secondary metabolites biosynthesis, transport and catabolism	2%	1%	2%
General function prediction only	14%	6%	10%
Function unknown	10%	23%	27%

Bold rows indicate those were a significant difference exists within the unique core genomes

### Metabolism and niche adaptation

To explore the divide between the subspecies in terms of their metabolic capabilities and to highlight particular niche adaptations within the strains, MCL analysis was employed to compare the COG groupings based on function i.e. [G] carbohydrate transport and metabolism, [E] amino acid transport and metabolism and [I] lipid transport and metabolism. These COG groups are fundamental to niche adaptation as they provide an overview of a strain’s ability to metabolise different energy sources. They may also include key technological traits sought in strains utilised in the dairy niche where the majority of sequenced strains have been isolated. Until now, the focus of this study has been on chromosome specific traits, however, in order to gain an overall view of the total metabolic capabilities of a strain it is necessary to also consider extra-chromosomal encoded traits. Therefore, both chromosomally- and plasmid-encoded features were considered for the remainder of the comparative analysis.

**Fig. 4:**
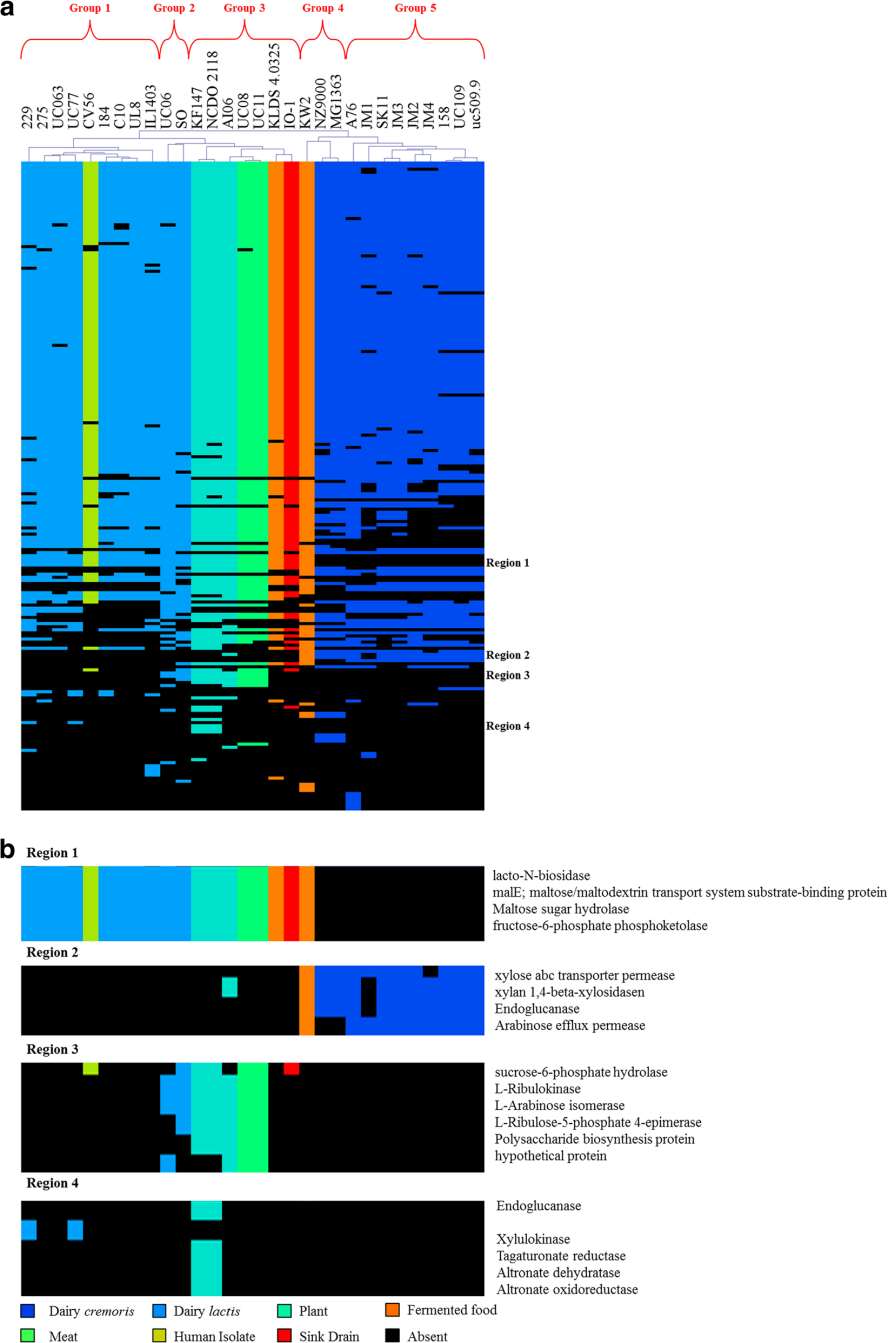
Carbohydrate utilisation and niche adaptation. **a** Hierarchical clustering analysis representing the presence/absence of gene families from COG group [G], carbohydrate transport and metabolism. Gene clusters are indicated based on the hierarchical tree, top. Colour indications refer to the particular niche from which the *L. lactis* strain had been isolated. **b** Enlarged views of variable regions 1–4 from the HCL matrix, involved in maltose and xylose transport and metabolism, and pentose and glucoronate interconversions

MCL analysis of COG [G] functions (genes involved in carbohydrate transport and metabolism) across all 30 isolates resulted in a gene presence/absence matrix displaying five groupings specific to niche environments (Fig. 4). The majority of analysed lactococcal genome sequences are derived from isolates from the dairy niche, where the most important adaptation is the ability to ferment lactose, facilitated by the products of the plasmid-borne *lac* operon, which consists of the *lacABCDEFGX* genes [8, 9]*.* The complete *lac* operon was identified in all subsp. *cremoris* strains isolated from the dairy niche except for the plasmid-free strains MG1363 and its derivative NZ9000. However, MG1363 has previously been shown to metabolise lactose due to the activity of a cellobiose-specific phosphotransferase system (PTS), which can act as an alternative lactose utilisation pathway under glucose starvation conditions [30]. The complete *lac* operon was also identified in six of the 11 subsp. *lactis* dairy isolates, yet not in the remaining five (strains 184, C10, UL8 and IL1403), of which *L. lactis* IL1403 is known to be a plasmid-cured strain [31]. When strains C10 and UL8 were inoculated in 10% RSM (reconstituted skimmed milk), they displayed no signs of growth or acidification, which is consistent with the observed absence of the *lac* operon. However, in the case of strain 184, growth on lactose is still observed, which can be explained by the presence of the cellobiose-specific phosphotransferase system (PTS), similar to the situation in MG1363 [30]. Interestingly, while all dairy-derived *cremoris* strains form a single cluster based on genes involved in carbohydrate metabolism with the exception of the laboratory strains MG1363 and NZ9000 (Group 4), all dairy-derived *lactis* strains (Group 1) with the exception of strains SO and UC06 (Group 2) form a single separate cluster to their *cremoris* counterparts based on carbohydrate utilisation. The only human isolate of *L. lactis* included in our analysis is also contained within Group 1. Differentiating factors, such as the clusters responsible for maltose utilisation found in all *lactis* strains and non-dairy *cremoris* strains, and for xylose metabolism as observed in all *cremoris* strains (with the exception of JM1), yet not present in *lactis* strains, contribute to this division (Fig. 4b). A major bifurcation in the HCL matrix between the two constituent subspecies was also observed, mirroring that of the supertree (Fig. 1b). Furthermore, the observed hierarchical groupings for the *cremoris* subspecies (Groups 4–5) closely relate to those observed in the supertree. Conversely, the observed groupings of the *lactis* subspecies (Groups 1–3) differ from those observed in the supertree as their increased metabolic abilities in terms of carbohydrate utilisation are not shown in the supertree which is built on conserved orthologous proteins. The clustering in the matrix is based on the absence/presence of proteins and as such none of these differences are included in the supertree.

The genomes of *L. lactis* UC08 and UC11 represent the only two complete lactococcal genome sequences isolated from fermented meat. In this analysis, these strains clustered closely with those derived from non-dairy sources, particularly plant-derived strains based on carbohydrate metabolism (Group 3). Genes encoding functions involved in pentose and glucuronate interconversions are found exclusively in strains isolated from the plant and meat niches, and thus are not present in any other lactococcal strain. These sugars are generally not found in milk where the primary sugar source is lactose with only trace amounts of monosaccharides and oligosaccharides. The majority of strains examined in this study are dairy isolates and so it is plausible that these functions have been lost through reductive evolution in strains adapted to the rich growth media provided by the dairy environment.

Supplementing COG analysis with information obtained from KEGG (Kyoto encyclopaedia of genes and genomes) analysis, a full assessment of all major metabolic pathways present in *L. lactis* was undertaken (Additional file 4). In this case complete pathways for D-galacturonate degradation (KEGG accession: M00631) and beta-oxidation, acyl-CoA synthesis (KEGG accession: M00086) were exclusively identified in the plant-derived strains NCDO2118 and KF147.

It has previously been demonstrated that both *L. lactis* subsp. *cremoris* and subsp. *lactis* are capable of folate biosynthesis [32]. Interestingly, KEGG analysis showed all analysed subsp. *lactis* strains to lack a complete pathway for tetrahydrofolate biosynthesis (KEGG accession: M00126) which was found to be complete in all subsp. *cremoris* strains. In *cremoris* strains the pathway was found to consist of nine genes responsible for conversions from purine metabolism to folate, whereas in subsp. *lactis* strains, the *phoA* gene that encodes an alkaline phosphatase (E3.1.3.1), responsible for the conversion of 7,8-dihydroneopterin 3-triphosphate to dihyroneopterin, appears to be absent. This indicates that this step in tetrahydrofolate biosynthesis in subsp. *lactis* is performed by an alternative and as yet unidentified enzyme (in comparison to their *cremoris* counter-parts).

### Amino acid transport and metabolism

**Fig. 5:**
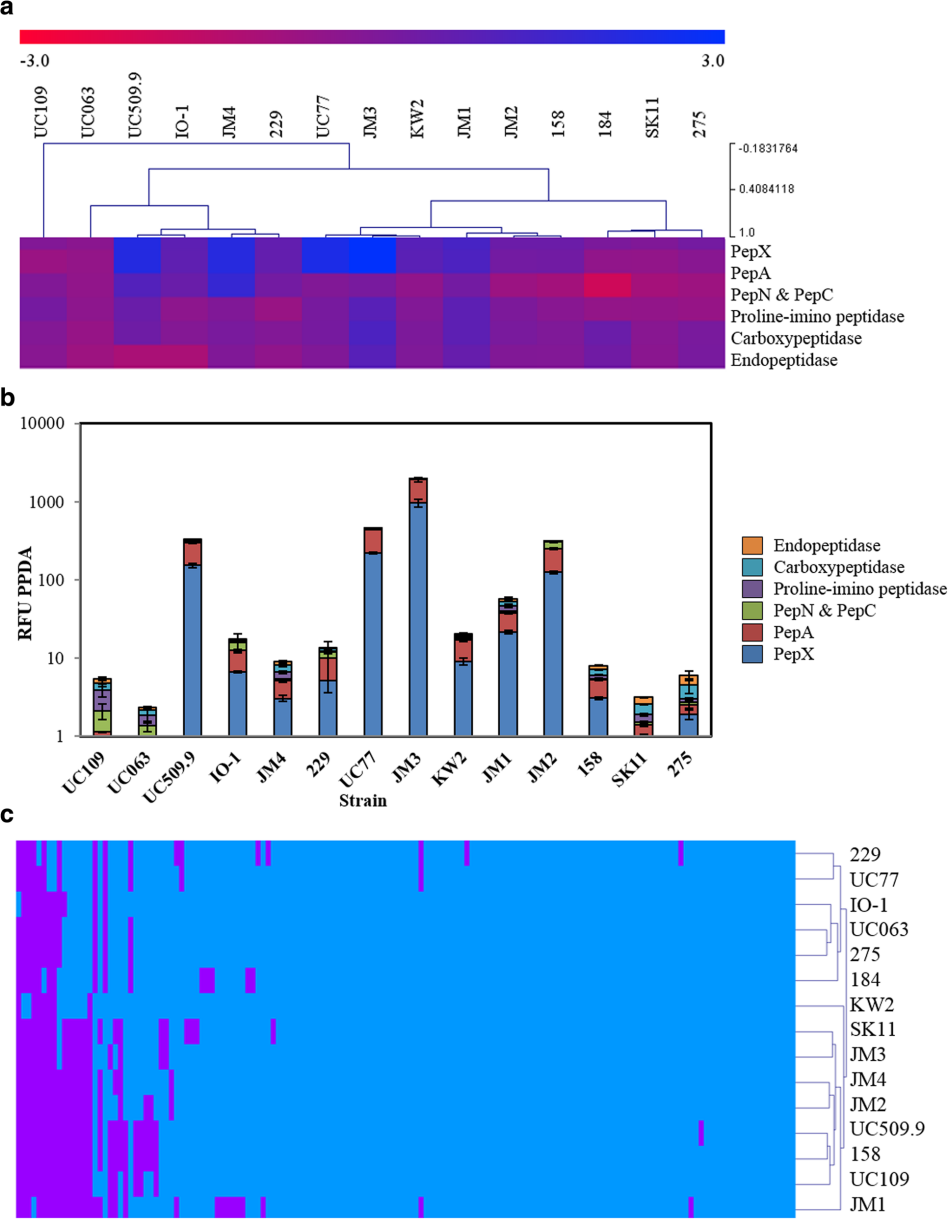
Peptide metabolism in *L. lactis.* **a** Level of PepX, PepA, PepN/C, Proline imino peptidase, endopeptidase and carboxypeptidase activity, expressed by *L. lactis* in log(RFU PPDA) where (1 RFU = the amount of μM of AMC released min^-1^ by 1 mg of protein). Strains are clustered based on activity red-blue indicating increased activity. **b** Total peptidase activity expressed by lactococcal strains for PepX, PepA, proline imino peptidase, endopeptidase, carboxy-peptidase and PepN/C in RFU PPDA (1 RFU = the amount of μM of AMC released min^-1^ by 1 mg of protein. **c** Hierarchical clustering analysis representing the presence/absence of gene families from COG group [E] amino acid transport and metabolism

Proteolysis (of casein) performed by *L. lactis* has been widely studied as it is considered to be an important technological trait in dairy lactococci due to its contribution to flavour in fermented dairy products such as cheese, as outlined by a number of reviews that detail this process [33–35]. The main categories of peptidases contributing to proteolysis in *L. lactis* are aminopeptidases, endopeptidases, di/tri-peptidases, proline peptidases, endopeptidases and carboxypeptidases. The majority of described peptidase-encoding genes represent monocistronic elements (e.g*. pepC*, *pepN* and *prtP*), while others are transcribed with genes apparently unrelated to proteolysis [36]. To assess the level of peptidase activity within *L. lactis*, both functional and genomic analyses were undertaken. Quantitative assays utilising fluorescently labelled substrates (see Materials and Methods section) were used to determine the activity levels of PepN/C, PepA, PepX, proline imino peptidase, carboxypeptidase and endopeptidase produced by each strain (Fig. 5a & b). The dominant peptidase activities expressed by each strain were those represented by the proline peptidase PepX and the aminopeptidases PepA and PepN/C. Interestingly, all of these peptidases are present in single-copy in each of the chromosomes, though the measured activity levels do vary considerably between strains. To ascertain a broader perspective on peptidase or amino acid digestion, an MCL analysis of COG group [E] amino acid transport and metabolism was performed (Fig. 5c). Clustering based on the presence or absence of genes involved in amino acid transport and metabolism resulted in two major groupings: the first composed of subsp. *lactis* strains and the second composed of *cremoris* strains indicating that the proteolytic system of these bacteria is distinct between and relatively well conserved within each subspecies.

**Fig. 6:**
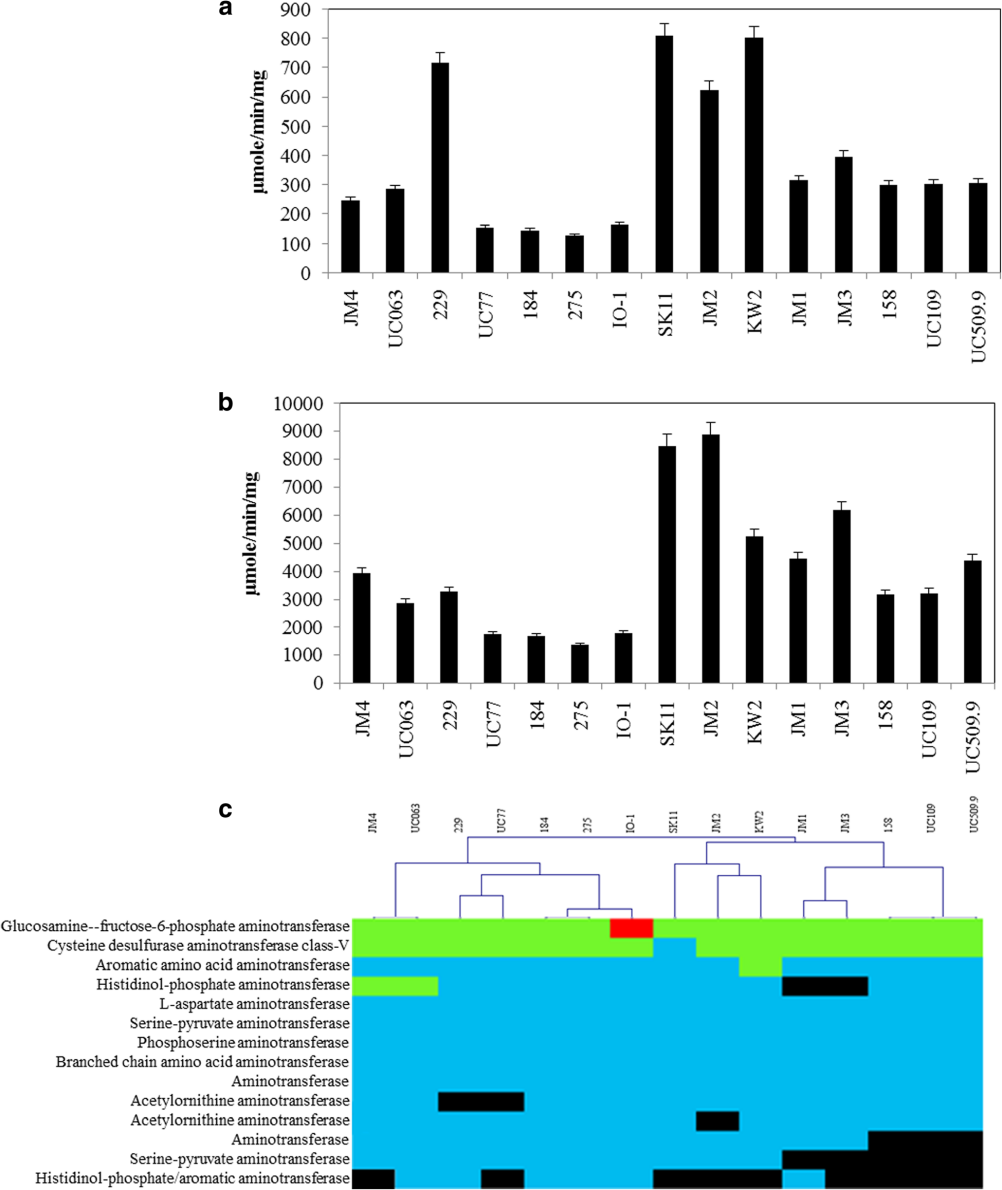
Aminotransferase activity in *L. lactis.* Amino acid transferase activities for (**a**) phenylalanine and (**b**) methionine. **c** Hierarchical clustering analysis representing the presence/absence of genes involved in aminotransferase activities. Copy number is indicated by colour; *red* (x3), *green* (x2), *blue* (single-copy) and *black* (absent)

Another important factor in assessing the proteolytic system of *Lactococcus* is the effect of amino acid transferases, which convert free amino acids to α-ketoacids. This is of particular importance when considering strains which may be used within the fermented food industry for the production of cheeses where aminotransferases contribute to flavour and aroma development [37]. As a high proportion of the current lactococcal dataset is currently composed of strains from the dairy niche, we assayed the strains for amino acid transferase activity against phenylalanine (aromatic amino acid) and methionine (sulphur amino acid), which are both common in milk and important in terms of cheese production. All strains demonstrated aminotransferase activity against phenylalanine (Fig. 6a) and a considerably higher level of activity against methionine (Fig. 6b). With the exception of *L. lactis* subsp. *cremoris* JM4, strains of the *cremoris* subspecies were shown to display significantly higher levels of aminotransferase activity compared to their *lactis* counterparts.

Markov clustering of aminotransferases in *L. lactis* strains was carried out and resulted in clades, which closely resemble the level of activity expressed by the constituent strains (Fig. 6c). Interestingly, strains SK11, JM2, and KW2, which exhibited the highest level of activity for both phenylalanine and methionine, did not encode the highest number of aminotransferases, and none of these strains specify a histidinol-phosphate/aromatic aminotransferase. Overall, both the peptidase and aminotransferase analyses indicated a very divergent proteolytic system between the two subspecies.

### Lipid transport and metabolism

**Fig. 7:**
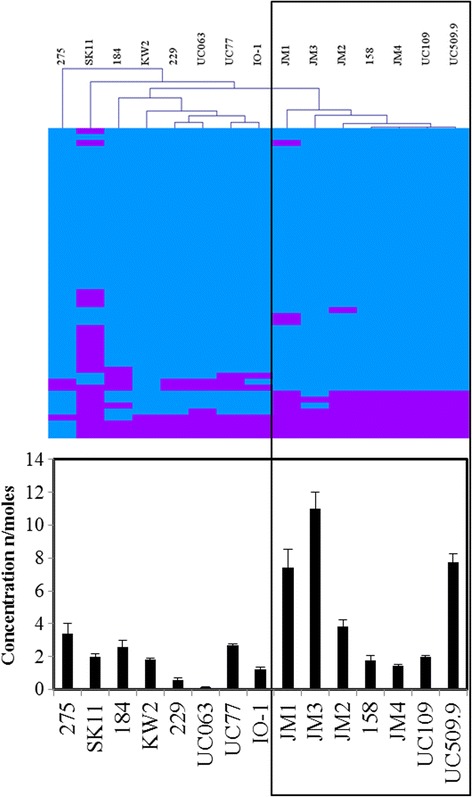
Lipid metabolism in *L. lactis.* Hierarchical clustering analysis representing the presence/absence of gene families from COG group [I] lipid transport and metabolism with associated histogram indicating level of short chain esterase activity of each constituent strain

MCL analysis combined with hierarchical clustering of COG group [I] (lipid transport and metabolism) revealed two main groups based on predicted lipolytic activity; the first was composed of both subsp. *lactis* and *cremoris* strains from mixed sources, while the second was composed exclusively of dairy *cremoris* strains, namely strains JM1, JM2, JM3, JM4, 158, UC109 and UC509.9. These strains encode a well-conserved lipolytic system, while lipolytic assays utilizing *p*-nitrophenyl-butyrate for the detection of short chain esterase activity revealed a trend showing higher expression of esterase activity by these strains compared to their subsp. *lactis* and non-dairy subsp. *cremoris* counterparts (Fig. 7).

### Plasmid integration

Bacterial adaptation relies heavily on the metabolic capabilities of the cell. In the case of *L. lactis* the most studied adaptations are those related to the dairy environment where reductive evolution or genome decay is observed among strains, a phenomenon which is believed to be due to repeated passaging in the nutrient-rich growth medium, milk [6, 7, 38]. As well as the loss of redundant metabolic functions to reduce energy- and resource-demanding systems in such a niche, the acquisition of new genetic information encoding traits that are advantageous to the host (in the particular niche) is often necessary. In *L. lactis,* the most notable example is adaptation to the dairy environment via the plasmid-borne *lac* operon, which allows for lactose utilisation as the primary sugar source, and the *prtP*-encoded protease and the *opp* operon responsible for amino acid/nitrogen acquisition from the milk protein casein. However, in some instances integration of such genetic features into the host’s chromosome may take place.

*In silico* based analysis of the chromosomes of 30 lactococcal isolates resulted in the identification of (1–6) integrated regions with significant (>90%) nucleotide identity to previously sequenced lactococcal plasmids (Additional file 5). The most notable of these putative integrations was the presence of the *opp* operon, originally identified as a plasmid-encoded trait in dairy *L. lactis* [39], conserved in the chromosomes of 24 out of 30 strains. The region shares (>90%) nucleotide identity with lactococcal plasmids pIL4, pQA549, pCIS8, pSK11L/SK11 plasmid 4, pVF50 and pGdh442. *L. lactis* MG1363 and its derivative *L. lactis* NZ9000 also harbour the associated *prtP* gene in the same integration site; however, it is integrated at approximately 680–690 Kbp on the chromosome. In one instance, for *L. lactis* SO, the associated *lac* operon, which controls lactose utilisation in the dairy niche, was detected on the chromosome, 20 Kbp downstream of the integrated *opp* operon and sharing significant homology with plasmids pCV56B, pSK08, pKF147A and pNCDO2118.

A number of other features that are typically observed among the plasmid complements of lactococci were identified in the chromosomes of the assessed strains including restriction-modification systems, conjugal transfer and mobilisation or *mob* genes, a partial lactococcin gene cluster (four instances) and a partial exopolysaccharide biosynthesis gene cluster (nine instances). The frequency of occurrence of these regions suggests that the total genetic complement of *L. lactis* is in a state of flux.

## Discussion

Recent advances in NGS technologies have made it easier to sequence a far greater number of high-quality bacterial genomes than ever before. In this study SMRT sequencing was applied for the complete sequencing of sixteen lactococcal genomes, more than doubling the existing number of publicly available, fully sequenced lactococcal genomes. The chromosomal features of *L. lactis* were assessed with particular emphasis on discerning the subspecies classification and niche adaptation of *L. lactis*.

Our analysis clearly identified a phylogenetic division between subspecies *lactis* and *cremoris*. This subspecies division was corroborated by hierarchical clustering based on both carbohydrate and amino acid metabolism, which indicates two main subgroups that correspond to each subspecies. Furthermore, for a number of conserved genes investigated in this study, a unique allelic type was observed for strains belonging to subsp. *lactis* and a separate allelic variant observed for strains belonging to subsp. *cremoris*. These observations support those made by Cavanagh and colleagues, who recently proposed a re-evaluation of the taxonomic group separating *L. lactis* into two distinct species *L. lactis* and *L. cremoris* based on ANI (average nucleotide identity) and TETRA (tetranucleotide frequency correlation coefficients) [16].

The percentage of pseudogenes within the sixteen lactococcal genomes sequenced in this study varies from 0.5 to 5.1%, when calculated as a percentage of the total number of coding sequences and is strain specific. Overall for the sixteen genomes sequenced in this study, this corresponds to an average pseudogene occurrence per strain of 1.76%, a percentage that is in line with the majority of prokaryotes [40]. The genomes of *L. lactis* subsp. *cremoris* were found to contain a higher number of pseudogenes in comparison to their *L. lactis* subsp. *lactis* counterparts, on average 100 per strain compared to 31 per strain, respectively. The vast majority of these strains are isolated from the dairy niche where genome decay and redundancy is widely reported [6, 38, 41], and believed to be due to continuous growth in milk. These genomes were also shown to contain a high number of prophages and transposable elements in agreement with Chopin and colleagues [5], and assumed to be the result of continued industrial pressures. Such prophages represent a risk factor, which warrants thorough assessment before introducing such strains into industrial fermentations. Conversely, the genomes of lactococcal strains isolated from both meat or plant environments displayed greater genetic variation and encode a greater number of metabolic pathways for the utilisation of a broader range of substrates compared to dairy-associated lactococci. The isolation of strains from these non-dairy sources may provide novel cultures for food fermentations and deliver desirable capabilities in terms of flavour and industrial robustness as dairy starter cultures.

COG analysis of *L. lactis* subsp. *cremoris* and *lactis* showed a higher proportion of genes involved in information processing and storage in *cremoris* strains and a higher proportion involved in metabolism in *lactis* strains, in the specific portions of the core genome the two subspecies do not share. This is in agreement with the generally observed reduced metabolic capabilities of subsp. *cremoris* strains, and highlights the reductive pressure through genome decay imposed on these (mostly) dairy-derived strains. This may also be conducive to the observed faster growth rate of *lactis* strains compared to their *cremoris* counterparts under milk fermentation conditions (Additional file 6). COG analysis was also utilised as a mechanism for functional genomic analysis in examining both peptide and lipid metabolism. It was determined that although strains can be genotypically clustered based on their subspecies and common niche, in agreement with a previous study [36], many of the peptidases for which functional assays are available exist in single copy in the majority of lactococcal genomes. Therefore, it may not always be possible to make the genotype-phenotype link without the involvement of transcriptome and/or metabolome-based studies to support the data. Interestingly, both peptidase and aminotransferase analyses indicated a very divergent proteolytic system between the two subspecies and relatively well conserved within each subspecies.

Niche adaptation also relies heavily on the acquisition of new metabolic capabilities as well as the loss of unnecessary functions. The introduction of niche-specific adaptations via plasmid acquisition, such as lactose and citrate metabolism has been extensively studied in *L. lactis* in view of their role in dairy niche adaptation [4, 8–10, 42], however, chromosomal adaptations are largely under-represented by comparison. Interestingly, the division between plasmid- and chromosome-based traits is becoming less clear as multiple integration events within the lactococcal chromosome suggests a more fluid genome than previously thought [4].

## Conclusions

In conclusion, the sequencing of 16 novel lactococcal isolates has doubled the number of complete finished quality lactococcal genomes available and allowed for large-scale comparative analysis of the complete metabolic systems of the taxon. Analysis of the two lactococcal subspecies revealed unique allelic subtypes for many of the conserved genes within each subspecies raising the question of their taxonomic placement and if the two subspecies should be redefined as separate species. Niche adaptation appears to play a significant part in governing the genetic content of each constituent strain, while genome decay and redundancy in the dairy niche is also widely observed. The deduced pan-genome of *L. lactis* is apparently closed, indicating that the representatives of this analysis are sufficient to fully describe the genetic diversity of the taxon.

## Methods

### Genome sequencing

All genomes sequenced in this study are dairy isolates of *L. lactis* subsp. *lactis* and subsp. *cremoris*, with the exception of *L. lactis* subsp. *lactis* UC08 and UC11, which were isolated from fermented meat products (Table 1). Chromosomal DNA from *L. lactis* subsp. *cremoris* JM1, JM2, JM3 and JM4 was isolated as previously described [43]. Chromosomal DNA extraction from *L. lactis* subsp. *cremoris* 158, UC109, *L. lactis* subsp. *lactis* UC11, C10, UL8 UC08, 275, UC063, UC06 184, 229 and UC77 was performed by commercial sequencing service providers GATC Biotech Ltd. (Germany).

SMRT sequencing was performed on a Pacific Biosciences RS II sequencing platform (executed by GATC Biotech Ltd., Germany). *De novo* genome assemblies were performed using the Pacific Biosciences SMRTPortal analysis platform (version 2.3.1), utilizing the RS_HGAP_Assembly.2 protocol. Remaining low quality regions or sequencing conflicts were resolved by primer walking and Sanger sequencing of PCR products (through sequence service proider Eurofins MWG Operon, (Germany).

### General feature predictions

After final genome assembly, Open Reading Frame (ORF) [or coding sequence (CDS)] prediction was performed with Prodigal v2.5 prediction software [44] and confirmed using BLASTX v2.2.26 alignments [28]. ORFs were automatically annotated using BLASTP v2.2.26 [28] analysis against the non-redundant protein databases curated by the National Centre for Biotechnology Information (NCBI) [45]. Following automatic annotation, ORFs were manually curated using Artemis v16 genome browser and annotation tool [46]. The software tool was used for the combination and inspection of ORF-identification results, for adjustment of start codons (where necessary), and for the identification of pseudogenes. Finally ORF annotations were refined further where required using alternative functional searches using Pfam [47], HHpred [48], PHAST [49] and Uniprot/EMBL [50].

Transfer RNA (tRNA) and ribosomal RNA (rRNA) genes were predicted using tRNA-scan-SE v1.4 [51] and RNAmmer v1.2 [52], respectively. Predicted RNA encoding genes were manually added to each genome using Artemis v16.

### Comparative genomics

The Mauve alignment tool was employed in order to perform whole genome alignments at the nucleotide level, and to explore synteny within the genomes and identify potential integration sites [53]. Genome synteny was explored and dotplots generated using Geopard v1.40 [54]. All sequence comparisons at the protein level were performed via all-against-all, bi-directional BLAST alignments [28]. An alignment cut-off value of; E-value 0.0001, >30% amino acid identity across 80% of the sequence length was used. For analysis and clustering of these results, the Markov Clustering Algorithm (MCL) was implemented in the mclblastline pipeline v12-0678 [29]. To further analyse genome-encoded functions, the protein complement was categorised based on COG (clusters of orthologous groups) assignments [55]. Metabolic pathways encoded by *L. lactis* strains were predicted and mapped using KEGG (Kyoto Encyclopaedia of Genes and Genomes) [56, 57]. Logo motifs were produced using WebLogo 3 [58].

### Phylogenetic analysis

The lactococcal supertree computation was performed by the BLAST-based comparative approach outlined above to identify a subset of 596 orthologous proteins. The subset was concatenated for each strain and an ungapped alignment was performed using MUSCLE v3.8.31 [59] with *Streptococcus thermophilus* LMG 18311 (Accession: CP000023) used as an outgroup. The phylogenetic tree was computed by the maximum-likelihood method in PhyML v3.0 and bootstrapped employing 1000 replicates [60]. The final tree file was visualised using ITOL (Interactive Tree of Life) [61]. 16S rRNA trees were prepared in MEGA6. Alignments were performed using MUSCLE. The evolutionary history was inferred by the Neighbour-joining method [62].

### Pan- and core-genome analysis

For the 30 available lactococcal genomes in this study, PGAP v1.0 [27] was used to perform the pan-genome analysis according to Heaps law pan-genome model [20]. The ORF content of each genome is organised in functional gene clusters via the Gene Family method. ORFs which produce an alignment with a minimum of 50% sequence identity across 50% of the gene/protein length are clustered and a pan/core genome profile is subsequently generated. The analysis is performed based solely on sequence identity and is not biased by functional annotation.

### Strain growth conditions and media

Bacterial strains used in this study are detailed in Table 1. *L. lactis* strains were routinely cultured at 30 °C in M17 broth (Oxoid) supplemented with 0.5% glucose/lactose without agitation. Alternatively strains where indicated, were grown in 10% RSM (reconstituted skimmed milk) at 30 °C without agitation.

### Intracellular enzymatic assays

Cells were prepared via a 1.5% inoculum into 10% RSM and grown overnight (16 hours) at 30 °C. Cells were then plated on M17 agarose supplemented with lactose to determine a viable plate count in cfu/ml. 50 ml of an overnight culture was added to 450 ml of borate buffer (0.05 M EDTA and 0.5 M borate pH8 with NaOH) and cells were collected by centrifugation (7000 rpm for 9 min). Cells were then washed in imidazole buffer (50 mmol/l imidazole and 10 mmol/l calcium chloride pH6.5) and pelleted by centrifugation (7000 rpm for 9 min). Cell pellet was re-suspended in 5 ml of lysis buffer (10 mM Tris-HCL, 50 mM CaCl_2_, 300 mM NaCl, 10 mM imidazole, 25 mg/ml of lysozyme, pH 7.5). Cells were then sonicated five times (30 s each) with 30 s on ice in between each sonication, after which cell debris was removed by centrifugation (15,000 rpm for 25 min at 4 °C). The resulting supernatant was then quantified for peptide/aminotransferase/esterase activity.

Detection of specific peptidase activities was conducted by fluorescence using 7-amino-4-methyl coumarin (AMC) coupled peptidase substrates; H-Lys-AMC.acetate (Lys-AMC) PepN and PepC, H-Asp (AMC)-OH (Asp-AMC) PepA, H-Pro-AMC.HBr (Pro-AMC) Proline imino peptidase, H-Gly-Pro-AMC. HBr (Gly-Pro-AMC) PepX, N-Suc-Gly-Pro-Leu-Gly-Pro-AMC (Gly-Pro-Leu-Gly-Pro-AMC) Endopeptidase and CBZ-Gly-Pro-AMC (Z-Gly-Pro-AMC) Carboxypeptidase, sourced from Bachem AG through VWR Ireland. The protocol was performed as described by Kato and colleagues [63], with the exception of reduced volumes for high throughput screening in 96-well plates. Released fluorescence was measured on a SpectraMax M3 Multi-Mode Microplate Reader from Molecular Devices. Enzyme activity was calculated in RFU PPDA (1 RFU = the amount of uM of AMC released min^-1^ by 1 mg of protein).

Amino acid transferase activity was determined (for Phe and Met) as previously described by Cavanagh and colleagues [16]. The final absorbance was read at wavelength, 300 nm in triplicate on a DU Series 730 spectrophotometer from Beckman Coulter, blanking the machine between each measurement. Standard curves were prepared for phenylalanine and methionine using phenylpyruvate and α-ketomethylthiobutyrate respectively. Amino acid transferase activity was then expressed as micromoles per minute per milligram of protein.

Detection of short chain esterase activity was conducted via a spectrophotometric assay as previously described [64], utilising *p*-nitrophenyl butyrate as a substrate. Absorbance was measured on a DU Series 730 spectrophotometer from Beckman Coulter. All activities measured were normalised for each strain based on cell count.

### Nucleotide sequence accession numbers

*L. lactis* subsp. *lactis* Il1403 AE005176, *L. lactis* subsp. *lactis* KF147 CP001834, *L. lactis* subsp. *lactis* CV56 CP002365, *L. lactis* subsp. *lactis* IO-1 AP012281, *L. lactis* subsp. *lactis* KLDS 4.0325 CP006766, *L. lactis* subsp. *lactis* NCDO 2118 CP009054, *L. lactis* subsp. *lactis* SO CP010050, *L. lactis* subsp. *lactis* AI06 CP009472, *L. lactis* subsp. *lactis* 184 CP015895, *L. lactis* subsp. *lactis* 229 CP015896, *L. lactis* subsp. *lactis* 275 CP015897, *L. lactis* subsp. *lactis* UC06 CP015902, *L. lactis* subsp. *lactis* UC08 CP015903, *L. lactis* subsp. *lactis* UC11 CP015904, *L. lactis* subsp. *lactis* UC063 CP015905, *L. lactis* subsp. *lactis* UC77 CP015906, *L. lactis* subsp. *lactis* UL8 CP015908, *L. lactis* subsp. *lactis* C10 CP015898, *L. lactis* subsp. *cremoris* SK11 CP000425, *L. lactis* subsp. *cremoris* MG1363 AM406671, *L. lactis* subsp. *cremoris* NZ9000 CP002094, *L. lactis* subsp. *cremoris* A76 CP003132, *L. lactis* subsp. *cremoris* UC509.9 CP003157, *L. lactis* subsp. *cremoris* KW2 CP004884, *L. lactis* subsp. *cremoris* 158 CP015894, *L. lactis* subsp. *cremoris* UC109 CP015907, *L. lactis* subsp. *cremoris* JM1 CP015899, *L. lactis* subsp. *cremoris* JM2 CP015900, *L. lactis* subsp. *cremoris* JM3 CP015901, *L. lactis* subsp. *cremoris* JM4 CP015909 and *S. thermophilus* LMG 18311 CP000023.

## Additional files

Additional file 1: Phylogenetic analysis of *L. lactis* housekeeping genes. Unrooted bootstrapped (x 100 replicates) maximum likelihood trees of; [A] *radA*, [B] *radC*, [C] *ssbA*, [D] *recQ* and [E] *recX*. Trees are coloured in accordance with subspecies type. (PPTX 116 kb)

Additional file 2: Whole genome nucleotide dotplots. Whole genome nucleotide alignments of thirty fully sequenced *L. lactis* genomes. Alignments 1(red)-18 represent subsp. *lactis* genomes. Alignments 19(black)-30 represent subsp. *cremoris* genomes. (PPTX 1744 kb)

Additional file 3: Unique gene family list. List of locus tag identifiers and predicted functions of unique gene families identified by MCL clustering. (XLSX 24 kb)

Additional file 4: KEGG ontologies. Overview of the presence or absence of complete intact KEGG modules in *Lactococcus*. (XLSX 17 kb)

Additional file 5: Plasmid integrations. Table detailing chromosomal regions sharing significant homology to previously sequenced lactococcal plasmids. (XLSX 15 kb)

Additional file 6: Pearce assay growth curves. Pearce assay growth curves of representative strains; [A] *L. lactis* subsp. *cremoris* JM1, [B] *L. lactis* subsp. *cremoris* JM4, [C] *L. lactis* subsp. *cremoris* JM3, [D] *L. lactis* subsp. *cremoris* JM2, [E] *L. lactis* subsp. *lactis* 229, [F] *L. lactis* subsp. *lactis* UC063, [G] *L. lactis* subsp. *cremoris* SK11, [H] *L. lactis* subsp. *cremoris* UC109, [I] *L. lactis* subsp. *cremoris* 158, [J] *L. lactis* subsp. *lactis* UC77, [K] *L. lactis* subsp. *lactis* 275, [L] *L. lactis* subsp. *lactis* 184. Black lines represent growth under Pearce assay conditions (Temperature: 32 °C for 70 min, 32–38 °C for 30 min, 38 °C for 160 min, 32 °C for 40 min). Red (subsp. *cremoris*) and blue (subsp. *lactis*) lines represent controls grown at 30 °C for 300 min. (PPTX 96 kb)

Additional file 7: Multilocus supertree orthologues and 16S rRNA genes list. List of locus tag identifiers and predicted functions of 596 orthologous protein sequences which were concatenated to generate the multilocus supertree and list of locus tag identifiers used to generate the 16S rRNA tree. (XLSX 34 kb)

## Abbreviations

BLAST: Basic alignment search tool
CDS: Coding sequence
COG: Cluster of orthologous groupings
HCL: Hierarchical clustering
ITOL: Interactive tree of life
MCL: Markov Clustering Algorithm
NGS: Next generation sequencing
ORF: Open Reading Frame
SMRT: Single molecule real time sequencing
