# Supplementary figures and images for: Comparative and functional genomics of the Lactococcus lactis taxon; insights into evolution and niche adaptation
Source: BMC Genomics. 2017 Mar 29;18:267. doi: 10.1186/s12864-017-3650-5 (PMC5372332; doi:10.1186/s12864-017-3650-5)

## Slide 1
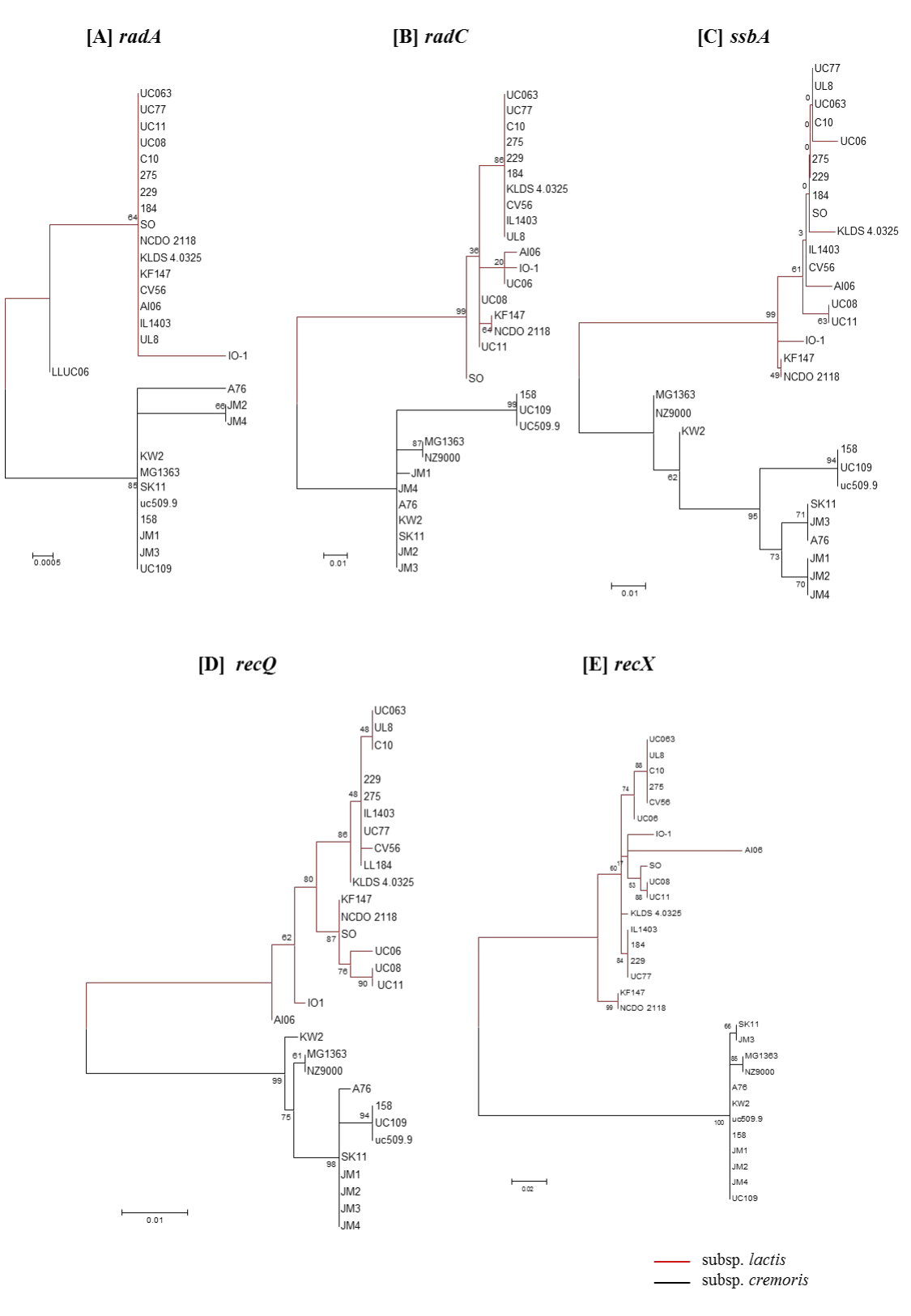

Supplement: Supplementary file 1 — Phylogenetic analysis of L. lactis housekeeping genes. Unrooted bootstrapped (x 100 replicates) maximum likelihood trees of; [A] radA, [B] radC, [C] ssbA, [D] recQ and [E] recX. Trees are coloured in accordance with subspecies type. (PPTX 116 kb) [file 12864_2017_3650_MOESM1_ESM.pptx]

## Slide 1
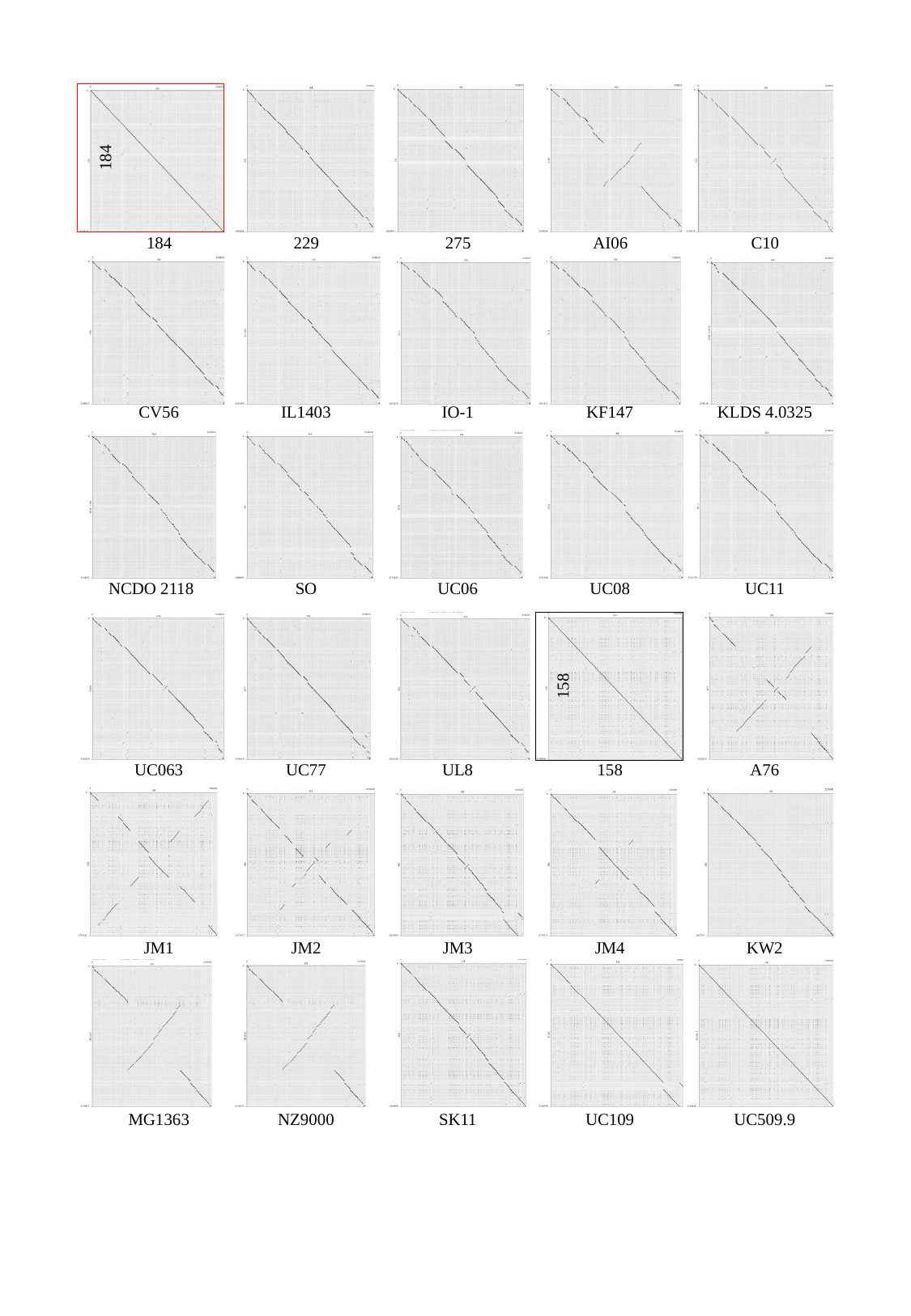

184
184
229
275
AI06
C10
CV56
IL1403
IO-1
KF147
KLDS 4.0325
NCDO 2118
SO
UC06
UC08
UC11
158
UC063
UC77
UL8
158
A76
JM1
JM2
JM3
JM4
KW2
MG1363
NZ9000
SK11
UC109
UC509.9

Supplement: Supplementary file 2 — Whole genome nucleotide dotplots. Whole genome nucleotide alignments of thirty fully sequenced L. lactis genomes. Alignments 1(red)-18 represent subsp. lactis genomes. Alignments 19(black)-30 represent subsp. cremoris genomes. (PPTX 1744 kb) [file 12864_2017_3650_MOESM2_ESM.pptx]
