# Supplementary material for: Comparative and functional genomics of the Lactococcus lactis taxon; insights into evolution and niche adaptation
Source: BMC Genomics. 2017 Mar 29;18:267. doi: 10.1186/s12864-017-3650-5 (PMC5372332; doi:10.1186/s12864-017-3650-5)
Supplement: Supplementary file 6 — Pearce assay growth curves. Pearce assay growth curves of representative strains; [A] L. lactis subsp. cremoris JM1, [B] L. lactis subsp. cremoris JM4, [C] L. lactis subsp. cremoris JM3, [D] L. lactis subsp. cremoris JM2, [E] L. lactis subsp. lactis 229, [F] L. lactis subsp. lactis UC063, [G] L. lactis subsp. cremoris SK11, [H] L. lactis subsp. cremoris UC109, [I] L. lactis subsp. cremoris 158, [J] L. lactis subsp. lactis UC77, [K] L. lactis subsp. lactis 275, [L] L. lactis subsp. lactis 184. Black lines represent growth under Pearce assay conditions (Temperature: 32 °C for 70 min, 32–38 °C for 30 min, 38 °C for 160 min, 32 °C for 40 min). Red (subsp. cremoris) and blue (subsp. lactis) lines represent controls grown at 30 °C for 300 min. (PPTX 96 kb) [file 12864_2017_3650_MOESM6_ESM.pptx]

## Slide 1
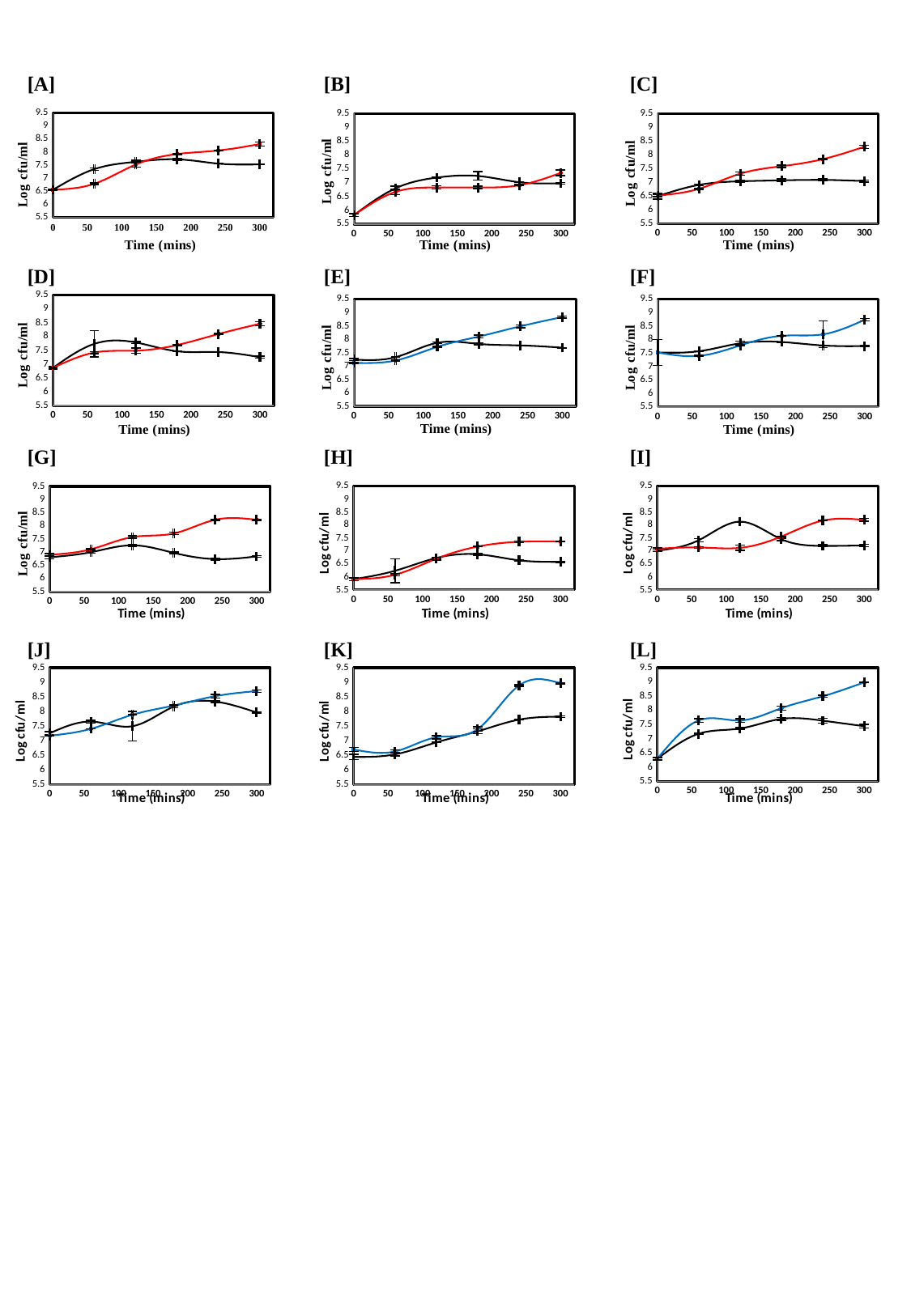

[A]
[B]
[C]
### Chart
| Category | | LF100P |
|---|---|---|
### Chart
| Category | | LF101P |
|---|---|---|
### Chart
| Category | | LF102P |
|---|---|---|[D]
[E]
[F]
### Chart
| Category | | LF106P |
|---|---|---|
### Chart
| Category | | |
|---|---|---|
### Chart
| Category | | |
|---|---|---|[G]
[H]
[I]
### Chart
| Category | | |
|---|---|---|
### Chart
| Category | | |
|---|---|---|
### Chart
| Category | | |
|---|---|---|[J]
[K]
[L]
### Chart
| Category | | |
|---|---|---|
### Chart
| Category | | |
|---|---|---|
### Chart
| Category | | |
|---|---|---|
